# Supplementary material for: Hippocampal cells integrate past memory and present perception for the future
Source: PLoS Biol. 2020 Nov 18;18(11):e3000876. doi: 10.1371/journal.pbio.3000876 (PMC7673575; doi:10.1371/journal.pbio.3000876)
Supplement: S1 Text — CMP, constructive memory-perception. (DOCX) [file pbio.3000876.s001.docx]

**S1 Text. Training procedures for the CMP task.** In order to prevent the monkeys from learning an association between each combination of the item-cue and the background-cue and its corresponding target location directly through repetitive trials by positive reinforcement learning, we first trained the monkeys to learn the task rule of the CMP task using a preliminary stimulus set in the preliminary training before the final training using a main stimulus set. For the preliminary stimulus set, we used monochromatic simple-shaped objects (e.g., green cross, red heart) as item stimuli and a large disk with four monochrome colors in individual quadrants as the background stimulus (S7 Fig). The monkeys were required to touch the target location on the touch screen (3M^TM^ MicroTouch^TM^ Display M1700SS) according to the combination of item-cue and background-cue without any fixation requirement during the task. We used a fixed orientation (0°) of the background image as the background-cue during the initial period of the preliminary training (S3 Table). After the animals learned the combination between items and the locations on the 0° background, we presented the oriented-background image as background-cue. The orientation of the background image was randomly chosen from -90° to 90° (0.1° step). After they learned the CMP task using the preliminary stimulus set, we trained them to learn the main stimulus set for the recording (Fig 1a and S3 Table). As to the main stimulus set, we first trained the animals on set A with the 0° background-cue, and then trained with the varied orientations (-90° to 90°) of background cues. The same procedure was repeated for set B. We found the animals learned the varied orientations of background-cue for the main stimulus set, particularly the set B, faster than the preliminary stimulus set, which may imply they learned the task rule during the preliminary training and applied it to the main stimulus set. The relatively slow learning of the set A, compared with the set B, might be due to the additional learning of the complicated background image itself and the applicability of the task rule, learned by the preliminary stimulus set, to other stimulus sets. Finally, we trained the monkeys to answer the target location by saccade. Once the monkeys reached the criterion of 70% accuracy, we started the recording session.
