# Supplementary material for: Hippocampal cells integrate past memory and present perception for the future
Source: PLoS Biol. 2020 Nov 18;18(11):e3000876. doi: 10.1371/journal.pbio.3000876 (PMC7673575; doi:10.1371/journal.pbio.3000876)
Supplement: S2 Text — (DOCX) [file pbio.3000876.s002.docx]

**S2 Text. Examinations of neuronal responses to co-location stimuli and eye positions.** We found that item-selective neurons showed similar amplitudes of activities to item stimuli that were assigned to the same location relative to the background-cue stimulus, implying that the item-selective responses reflected to-be-retrieved location information. One potential caveat to this interpretation would be that the location signal of the item-selective neurons reflected animals’ eye positions rather than the item-location association memory although the animals were required to maintain fixation on the center. To examine this possibility, we first evaluated the co-location effects of item-cue stimuli on eye positions during the item-cue period when we collected spike-firing data of item-selective neurons. We grouped trials with two different item-cues sharing the same location into one type of co-location trials. We conducted one-way ANOVA with four types of co-location trials as the main factor for the recording session of each item-selective neuron, and found that 37% of recording sessions showed significant (*P* < 0.025, either horizontal or vertical) effects of co-location trials on eye position (item-selective neurons with recorded eye data, n = 89).

We then separated item-selective neurons into two groups according to the co-location effects of item-cue stimuli on eye positions during the recording sessions. If the location signal of the item-selective neuron was explained by the eye positions, the location signal should be stronger when the co-location of item-cue affects eye positions more. We examined whether or not the item-selective responses signaled the location information during the item-cue period by calculating the correlation coefficients between the responses to co-location stimuli. The correlation coefficients were even larger for the item-selective neurons sampled during the recording session without significant co-location effects on the eye positions (*r* = 0.82, median) than those with significant effects (*r* = 0.73). These results indicate that the location information exhibited by item-selective neurons could not be explained by the animals’ eye positions. Because of hardware defects, we failed to record eye positions during the early period of the experiment (47 of 136 item-selective neurons).
