# Supplementary material for: Hippocampal cells integrate past memory and present perception for the future
Source: PLoS Biol. 2020 Nov 18;18(11):e3000876. doi: 10.1371/journal.pbio.3000876 (PMC7673575; doi:10.1371/journal.pbio.3000876)
Supplement: S3 Text — (DOCX) [file pbio.3000876.s003.docx]

**S3 Text. Detection of task-related signals during the background-cue period.** To quantify neuronal responses during the background-cue period, we performed three-way nested ANOVA with co-location, background-cue, and target as the main factors for each neuron. Item-cues were nested under the co-location factor, and their effects were negligible during the background-cue period in all areas. In this analysis, we used data only in correct trials whose background-cues were -90°, 0°, and 90° because -45° and 45° background-cues bring about target positions (i.e., top, right, bottom, and left) that were different from those of the -90°, 0°, and 90° background-cues (top-right, bottom-right, bottom-left, and top-left). Because of the limited orientations of the background-cue, a co-location of the item-cue would theoretically bring about a bias for a target location (e.g., co-location I could result in top-right, bottom-right, and top-left, but not bottom-left target location). To test an effect of this confounding factor on the detection of target-selective activities, we applied the three-way nested ANOVA to responses during the item-cue period and found only a negligible number (1%) of neurons with significant (*P* < 0.01) target-selective activities during the item-cue period (c.f., 16% during the background-cue period). This result suggests that the confounding factor did not bring about a significant bias on the detection of target-selective activities in the present study.
