# Supplementary material for: Hippocampal cells integrate past memory and present perception for the future
Source: PLoS Biol. 2020 Nov 18;18(11):e3000876. doi: 10.1371/journal.pbio.3000876 (PMC7673575; doi:10.1371/journal.pbio.3000876)
Supplement: S1 Table — (DOCX) [file pbio.3000876.s004.docx]

|  | **Total** | **Monkey B** | **Monkey C** |
| --- | --- | --- | --- |
| **Recorded** | 456 | 247 | 209 |
| **I-Cue period** |  |  |  |
| **Item** | 136 | 66 | 70 |
| **B-Cue period** |  |  |  |
| **Co-location** | 66 | 33 | 33 |
| **Background** | 66 | 32 | 34 |
| ***Convergence*** | 32 (19) | 14 (6) | 18 (13) |
| **Target** | 72 | 39 | 33 |
| ***Transference*** | 23 | 7 | 16 |
| ***Targeting*** | 24 | 18 | 6 |
| ***Multiple*** | 18 (4) | 6 (2) | 12 (2) |

**S1 Table. Numbers of task-related neurons.** “Item” indicates neurons with an item effect during the item-cue (I-Cue) period (*P* < 0.01, one-way ANOVA). “Co-location”, “Background” and “Target” indicate neurons with co-location, background and target-location effects during the background-cue (B-Cue) period, respectively (*P* < 0.01, three-way nested ANOVA). “*Convergence*” indicates the neurons showing both co-location and background effects during the background-cue period. Numbers in parentheses indicate neurons with both co-location and background effects in at least one same bin. “*Transference*” indicates the neurons with target effect that showed significantly stronger responses to -90°/90° background-cue than those with 0° background-cue in trials with the preferred target location (*P* < 0.05, *t*-test, one-tailed) during 300 ms of time-bin centering on response peak-times. The response peak-time was determined in the trials with -90°/90° background-cue resulting the preferred target location for each target-selective neuron (median = 440 ms, n = 23 transference neurons). “*Targeting*” indicates neurons showing only a target effect (neither co-location nor background-cue effect) during the last 300 ms of the background-cue period. “*Multiple*” indicates neurons showing two or three of convergence, transference and targeting responses. Numbers in parentheses indicate neurons showing all three types of responses.
