# Supplementary material for: Hippocampal cells integrate past memory and present perception for the future
Source: PLoS Biol. 2020 Nov 18;18(11):e3000876. doi: 10.1371/journal.pbio.3000876 (PMC7673575; doi:10.1371/journal.pbio.3000876)
Supplement: S2 Table — (DOCX) [file pbio.3000876.s005.docx]

|  | **Total** | **Monkey B** | **Monkey C** |
| --- | --- | --- | --- |
| **Top-right** | 18 | 11 | 7 |
| **Bottom-right** | 24 | 11 | 13 |
| **Bottom-left** | 16 | 8 | 8 |
| **Top-left** | 14 | 9 | 5 |

**S2 Table. Numbers of target-selective neurons selective to each target location.** “Top-right”, “Bottom-right”, “Bottom-left” and “Top-left” indicate the target-selective neurons with preferred responses to top-right, bottom-right, bottom-left, and top-left target locations, respectively. Target-selective neurons of Monkey B and Monkey C were recorded from the left hemisphere and right hemisphere, respectively.
