# Supplementary material for: Hippocampal cells integrate past memory and present perception for the future
Source: PLoS Biol. 2020 Nov 18;18(11):e3000876. doi: 10.1371/journal.pbio.3000876 (PMC7673575; doi:10.1371/journal.pbio.3000876)
Supplement: S3 Table — CMP, constructive memory-perception. (DOCX) [file pbio.3000876.s006.docx]

|  | **Average** | **Monkey B** | **Monkey C** |
| --- | --- | --- | --- |
| **Preliminary stimulus set** |  |  |  |
| **0° background-cue** | 9 | 9 | 9 |
| **-90°~90° background-cue** | 42 | 39 | 45 |
| **Main stimulus set** |  |  |  |
| **Set A (0° background-cue)** | 16 | 20 | 12 |
| **Set A (-90°~90° background-cue)** | 35.5 | 33 | 38 |
| **Set B (0° background-cue)** | 4 | 4 | 4 |
| **Set B (-90°~90° background-cue)** | 15 | 16 | 14 |

**S3 Table. Numbers of training sessions for the CMP task.** Sessions (days) of each stimulus set (4 items) for the first 0° fixed background-cue condition and the second -90°~90° randomized background-cue condition (see also S1 Text).
