# Supplementary figures and images for: Hippocampal cells integrate past memory and present perception for the future
Source: PLoS Biol. 2020 Nov 18;18(11):e3000876. doi: 10.1371/journal.pbio.3000876 (PMC7673575; doi:10.1371/journal.pbio.3000876)

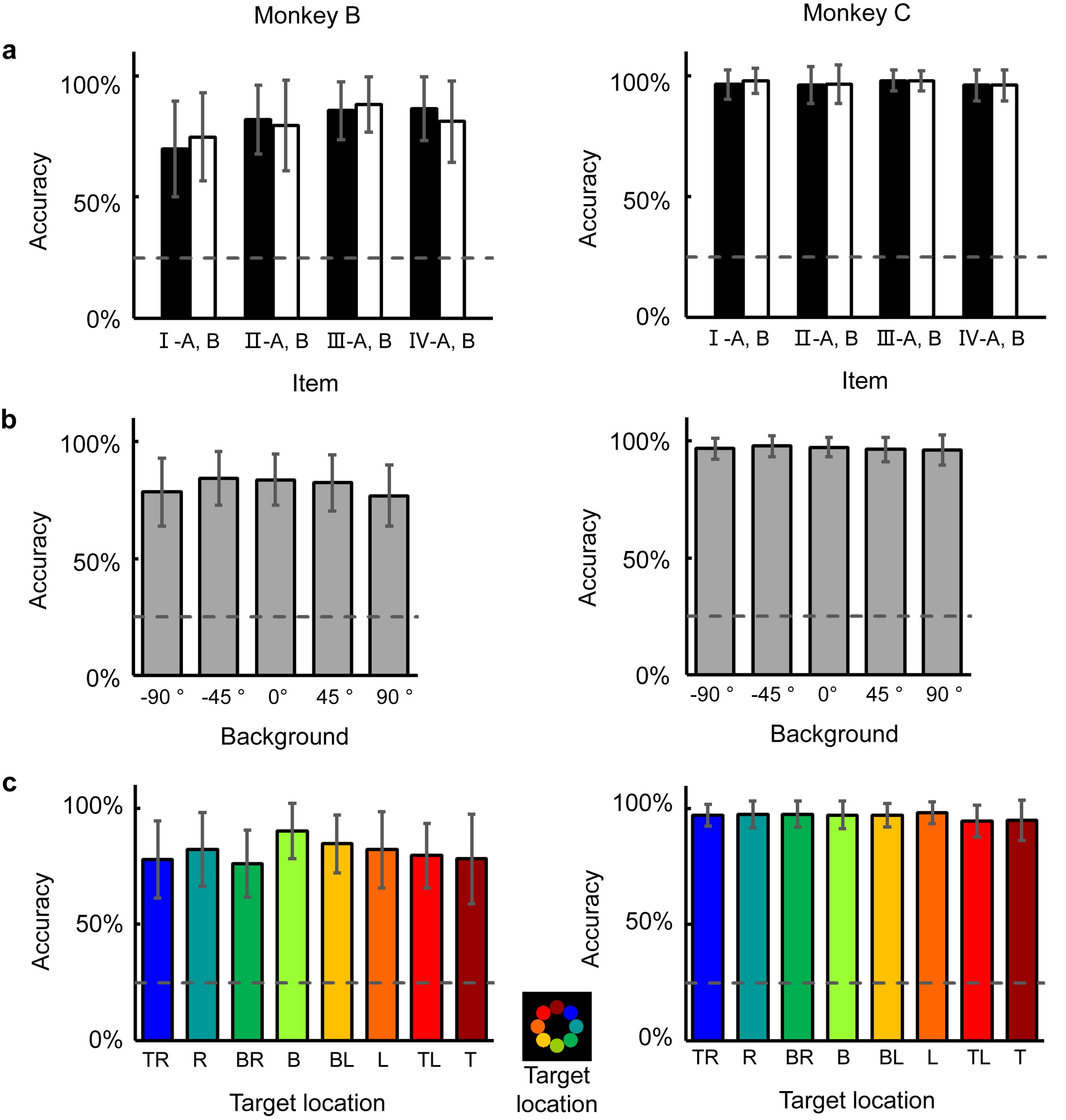

Supplement: S1 Fig — Performance during recording sessions (n = 179 for Monkey B, n = 158 for Monkey C). Error bar, standard deviation. Dashed line, chance level = 25%. (a) Performance for 8 item stimuli as item cue. Black bars, set A. White bars, set B. (b) Performance for 5 orientations of background cue. (c) Performance for 8 positions on the display as target locations. Source data are available in S2 Data. B, bottom; BL, bottom left; BR, bottom right; CMP, constructive memory-perception; L, left; R, right; T, top; TL, top left; TR, top right. (TIF) [file pbio.3000876.s007.tif]

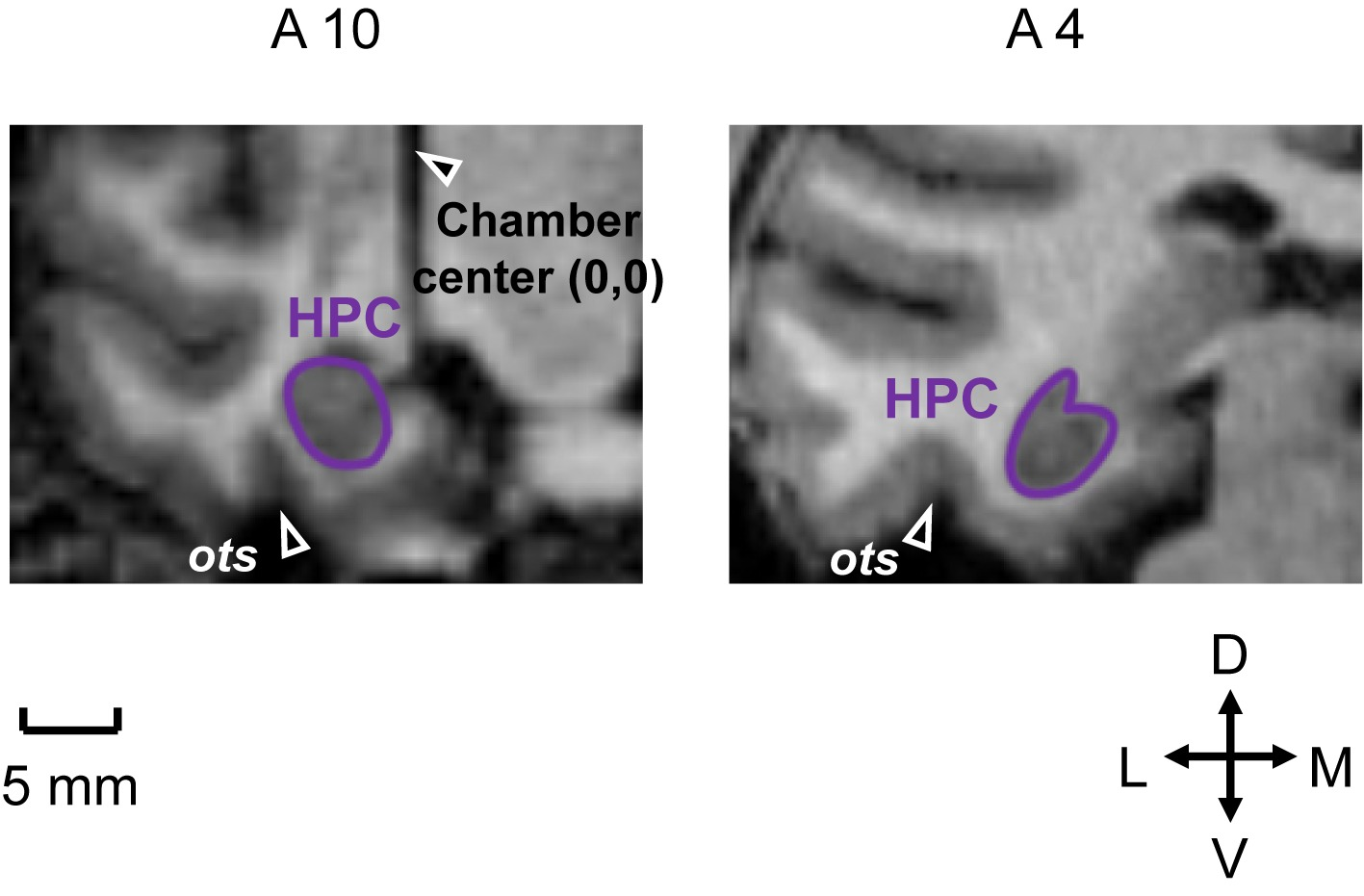

Supplement: S2 Fig — Magnetic resonance images corresponding to the coronal planes anterior 4 and 10 mm from the interaural line of monkey C (right hemisphere). The recording region is the HPC. A reference electrode implanted in the center of chamber was observed as a vertical line of shadow in the coronal plane at A10. D, dorsal; HPC, hippocampus; L, lateral. M, medial; ots, occipital temporal sulcus; V, ventral. (TIF) [file pbio.3000876.s008.tif]

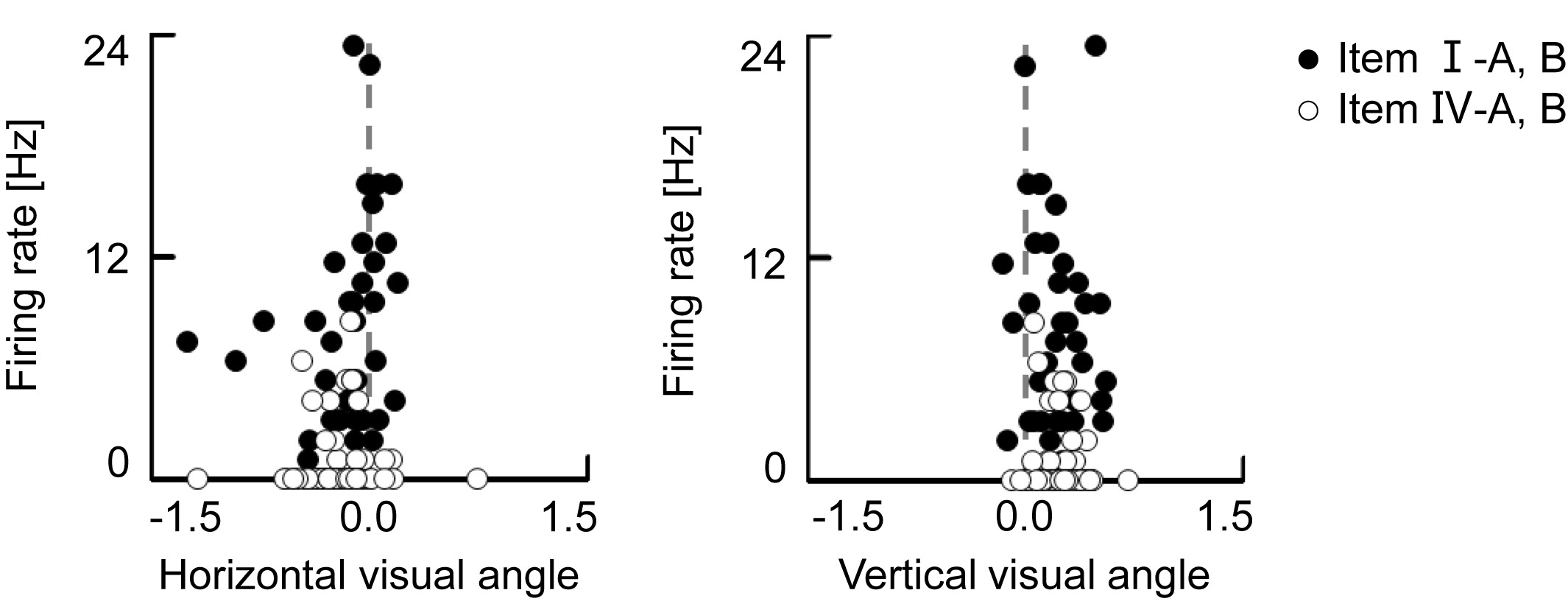

Supplement: S3 Fig — Firing rates plotted as a function of eye positions during the item-cue period for the neuron shown in Fig 2A and 2B. Each circle indicates 1 trial. Filled circles indicate trials with the best co-location stimuli as item cues. Open circles indicate trials with the worst co-location stimuli as item cues. The large overlaps were found in the distributions of the eye positions between the trials with the best and worst co-location items (P = 0.16 for horizontal, P = 0.25 for vertical, t test, 2-tailed), whereas distributions of the firing rates were significantly different between the 2 trial types (P < 0.0001). These results indicate that the item-selective responses shown in Fig 2 cannot be explained by the animal’s eye positions. Source data are available in S2 Data. (TIF) [file pbio.3000876.s009.tif]

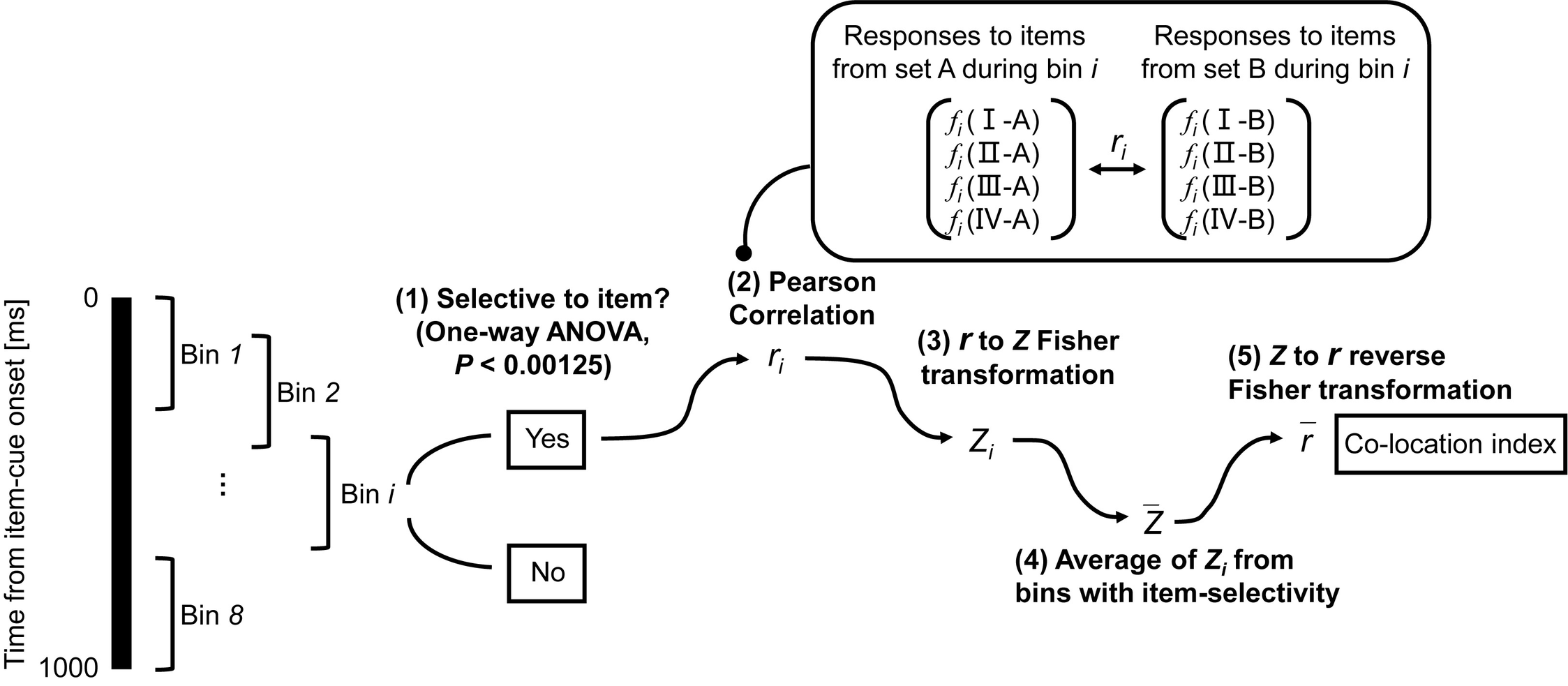

Supplement: S4 Fig — The co-location index was calculated for each neuron as following 5 steps. (1) The item-selectivity was examined in each of 8 consecutive 300-millisecond time-bins using the same threshold (i.e., P < 0.0125 for each time-bin, 1-way ANOVA) as that for the definition of item-selective neurons (P < 0.01, Bonferroni correction for 8 analysis time-bins). (2) If the time-bin showed a significant item-selectivity, we calculated the correlation coefficient (r) between the responses to items from set A and those from set B in the time-bin, and (3) then the r was transformed into Z. (4) We then averaged Z values across the significant time-bins and (5) re-transformed the averaged Z value into r¯ value as the co-location index of the neuron. (TIF) [file pbio.3000876.s010.tif]

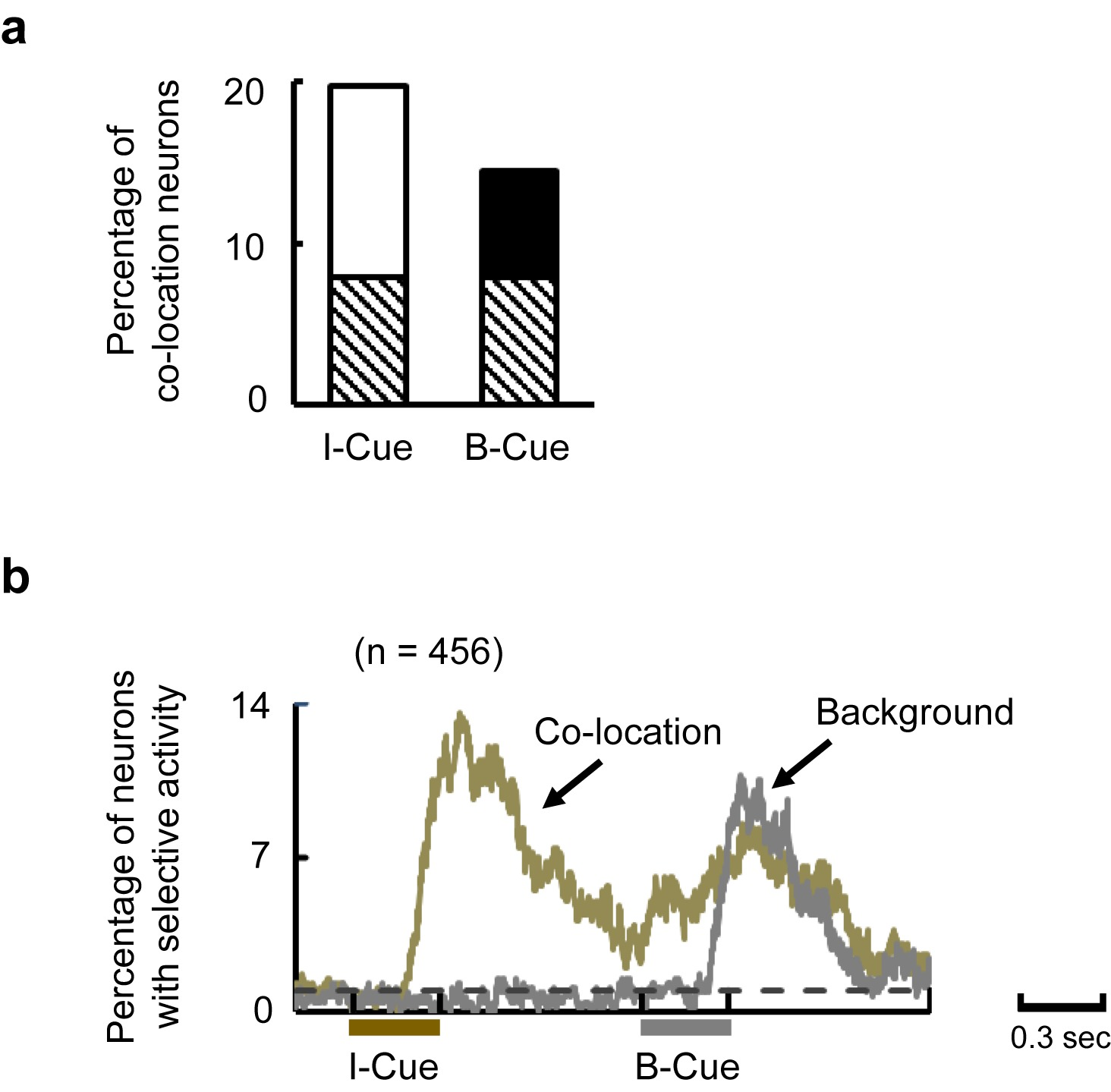

Supplement: S5 Fig — (a) Percentages of neurons showing a significant co-location effect (P < 0.01, 3-way nested ANOVA) during the item-cue (I-Cue) and background-cue (B-Cue) periods out of the recorded neurons (n = 456). Hatched area, neurons exhibiting co-location-selectivity during both periods. (b) Time courses of percentages of neurons showing significant co-location-selective activity and background-selective activity (100-millisecond time bin, P < 0.01, 3-way nested ANOVA, uncorrected) out of the recorded neurons (n = 456). Brown bar, presentation of the item-cue. Gray bar, presentation of the background cue. Dashed line, chance level = 1%. Source data are available in S2 Data. (TIF) [file pbio.3000876.s011.tif]

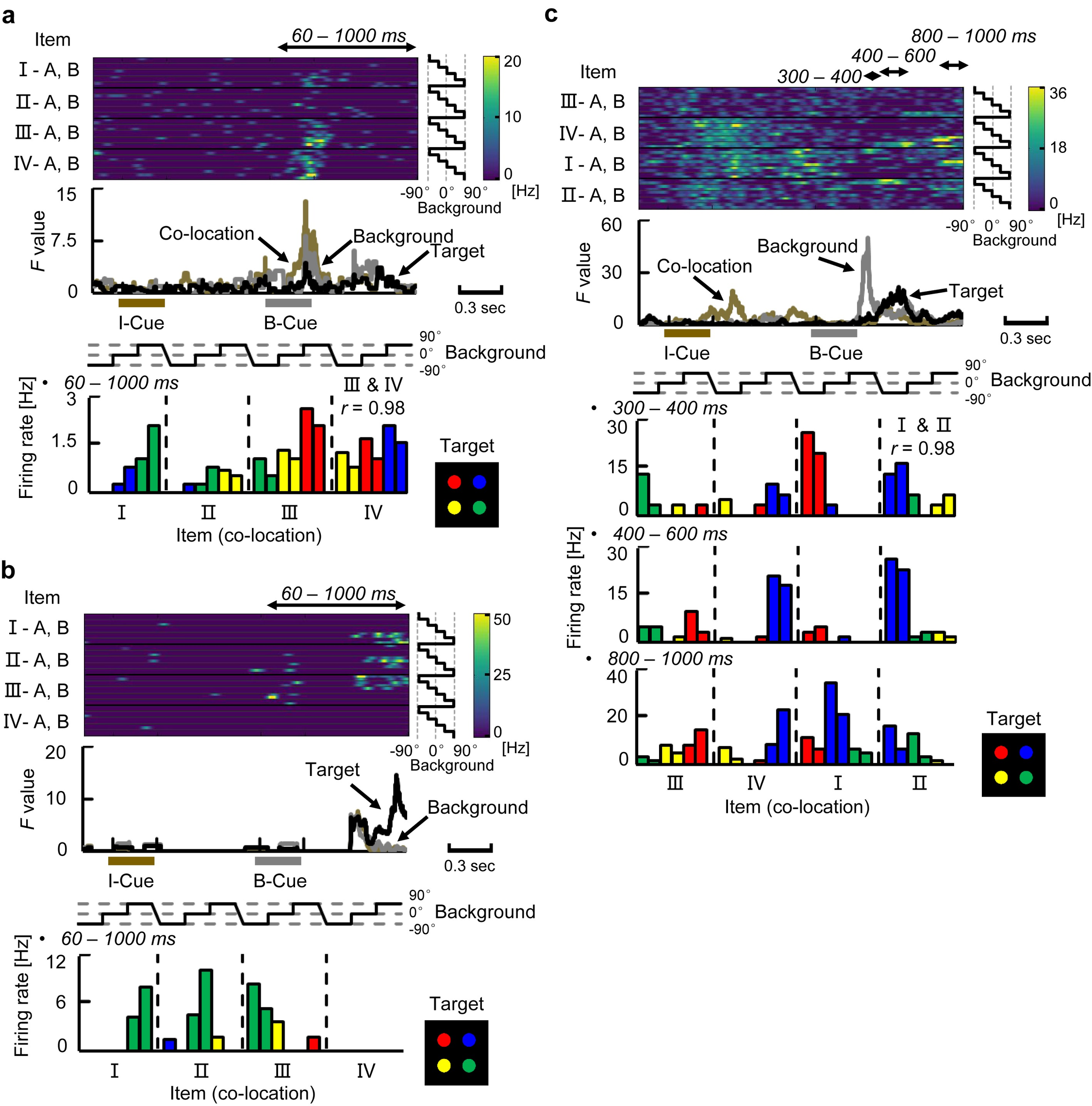

Supplement: S6 Fig — (a) Example neuron signaling co-location and background cue in a “convergent” manner. This neuron did not show item-cue selective responses during the item-cue period (P = 0.34, 1-way ANOVA), but it exhibited the co-location-selective responses during the background-cue period (P < 0.01, 3-way nested ANOVA). The background-selective responses were combined with the co-location-selective responses. The preferred orientation of the background-cue stimulus was 90° for this neuron across co-locations. The same format as Fig 5A. (b) Example neuron signaling a “targeting” location. This neuron did not show item-selective responses during the item-cue period (P = 0.81), but it exhibited the target-selective responses during the background-cue period (P < 0.0001). The best target location was bottom-right of display (green). (c) Example neuron that changed the activity patterns, showing multiple operations for the construction (i.e., convergence, transference, and targeting) during the background-cue period. This neuron showed item-selective responses during the item-cue period (P < 0.0001), and the preferred items in the item-cue period were I-A, I-B, IV-A, and IV-B. During 300–400 milliseconds after background-cue onset, the background-selective responses were combined with the co-location-selective responses, and the preferred orientation was −90° across co-locations (i.e., “convergence”). During 400–600 milliseconds after background-cue onset, this neuron exhibited strong responses only to the particular combinations of item cue and background cue that corresponded to the top-right target position (blue disk) (blue bars, II-A and II-B, −90° and IV-A and IV-B, 90°) (i.e., “transference”). During 800–1,000 milliseconds after background-cue onset, this neuron exhibited selective responses to the top-right target position regardless of item and background cues (i.e., “targeting”). Source data are available in S2 Data. (TIF) [file pbio.3000876.s012.tif]

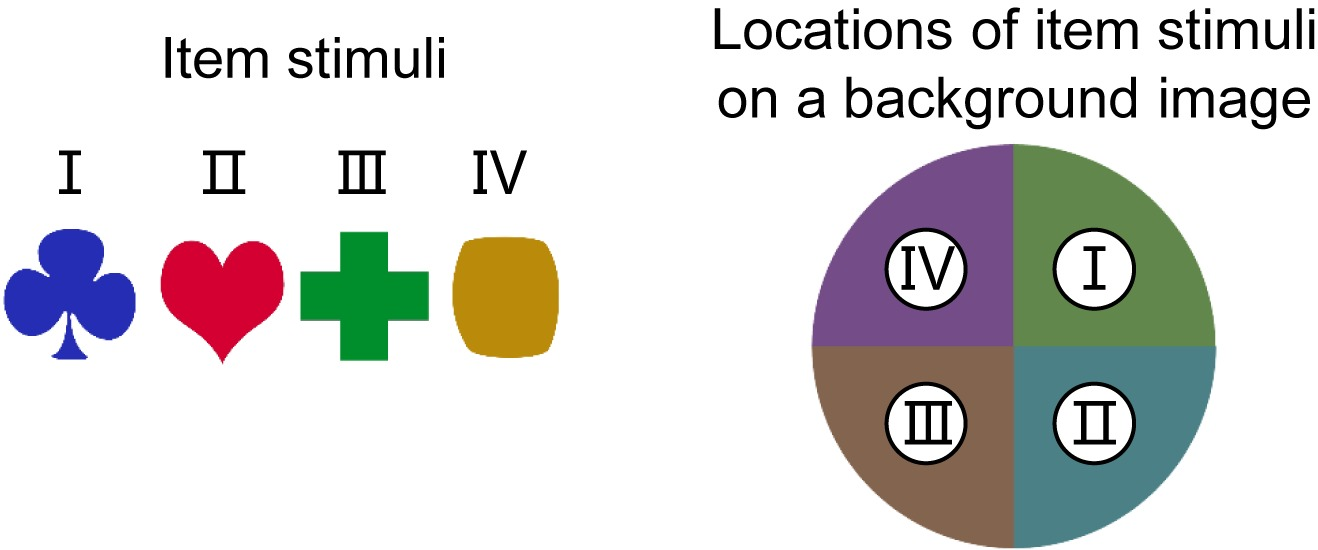

Supplement: S7 Fig — (Left) Simple shape objects with monochrome colors as item-cue stimuli. (Right) Large disk with 4 monochrome colors in individual quadrants as a background-cue stimulus. Each item stimulus was assigned to 1 location on the background image. CMP, constructive memory-perception. (TIF) [file pbio.3000876.s013.tif]
