# Supplementary material for: Hippocampal cells integrate past memory and present perception for the future
Source: PLoS Biol. 2020 Nov 18;18(11):e3000876. doi: 10.1371/journal.pbio.3000876 (PMC7673575; doi:10.1371/journal.pbio.3000876)
Supplement: Attachment — Submitted filename: Response Yang Naya.docx [file pbio.3000876.s016.docx]

**PBIOLOGY-D-20-00920R1**

**Responses to Reviewer #1:**

We thank the reviewer for the recognition of our work as well as the important suggestions and comments on the description of our manuscript. We addressed all the comments listed point-by-point and revised our manuscript accordingly.

***Comment 1:*** In the CMP task, the "transference" process from the retrieved location into a target location might be a type of "mental rotation", i.e., rotating the retrieved cue location in a mental image to the direction indicated by the background cue. Though the authors already discussed similar issues in lines 258-276, a brief statement discussing the relation of their data and mental rotation in HPC and/or related areas with some references, if possible, may be helpful for the readers.

***Response***: According to the present findings, the “transference” process may not reflect “mental rotation.” We found “convergence” process which combined the retrieved location and the background orientation into the single neurons’ activities, which held both signal contents. If we assume that the target location information was constructed from this convergent-type of activity reflecting the current background-cue information, which was already oriented, it would be reasonable to consider that the retrieved location was directly transferred to the target position rather than the “mental rotation.” This direct transfer may occur when a subject is familiar with a current environment, in which enough information would be provided to compute the target location accurately in the HPC. Conversely, when the environment is not enough familiar, the subject may recruit an active simulation like mental rotation, which would be supported by other cortical areas (Bellmund et al., 2016; Hawes et al., 2019; Lamm et al., 2007). We discussed the relation of the present data and mental rotation in the revised manuscript (Page 19, Lines 388-398).

***Comment 2:*** It might be helpful if the authors would present a summary graph or table of total proportions of the task-related specific neurons, i.e., neurons representing retrieved memory (Fig.2), neurons representing convergence (Fig.3), neurons representing retrieved and target locations (Fig.4), neurons constructing goal-directed information (showing transference effect) (Fig.5), and neurons showing multiple operations (Fig.5). This summary could help us to make an image of information processing of declarative memory in neurons and neuronal population levels in HPC. I expect that the numbers of these neurons are different in a systematic way and more discussion will be possible in future studies.

***Response***: We added the numbers of related neurons with their definitions in S1 Table.

***Comment 3:*** The authors reported that the signals of mnemonic information and perceptual information were combined at a single-neuron level in HPC. The mechanism of such convergence of different information for past and present to a single neuron is the great finding by the authors. On the other hand, as a conceptual assumption, convergence of different elementary information could be realized by co-activation of neurons related to elementary information alone. It might be helpful for me and the readers if the authors would add a brief discussion of how the mechanism of convergence to individual neurons (convergence neurons) is advantageous to realize the dynamic features of declarative memory.

***Response***: Thank you for the important comment. Yes, the convergence of different elementary information could be realized by co-activation of neurons related to elementary information alone. However, a receiver is necessary to detect the co-activation. Here, the important point might be how the two elementary information converge and construct a new information. In the present study, we found the convergent-type process, which held different elementary information at the single neuron level. We hypothesized that this convergent-type activity may be useful to compute a target location from the two input signals which have an internal relationship (e.g., item-cue and background-cue in the CMP task), which may equip us to answer a direction of a target from our current position/orientation even though we do not have a direct experience to travel between them. We discussed this point in the revised manuscript (Page 18, Lines 375-387).

**Responses to Reviewer #2:**

We thank the reviewer for the recognition of our work as well as the suggestions and comments on the analysis and description of our manuscript. We addressed all the comments listed point-by-point and revised our manuscript accordingly.

***Comment 1:*** They classified the target-selective neurons in two types: 'targeting-type' (Fig 4) and 'transferring-type' (Fig 5). The 'targeting-type' neurons were activated when the direction difference between the retrieved location (co-location) and target direction was 0 degree. On the other hand, the 'transferring-type' neurons were activated when the target directions were 90 and -90 degrees from the retrieved location. However, the descriptions of these two types are sometimes confusing in the manuscript. For example, the sentence "these (= item and target selective) neurons tended to show the preferred target locations that corresponded to the preferred co-locations relative to the 0° background-cue (default orientation), but not to the other orientations (Fig 4c)" sounds contradictory because the transferring-type neurons preferred the opposite. (This seems because the analyzed populations for 'transferring-type' and 'targeting-type' are different, but the reason why they selected the different populations for these two types is not clear.) More structural and comprehensive explanations and statistical analysis of these two types of neurons would help readers to understand the importance of the results. Also, adding the numbers of 'targeting-type' and 'transferring-type' neurons in S1 Table would be informative.

***Response***: The ‘targeting-type’ activity signaled the target location regardless of the co-locations of item-cues or the orientations of background-cues. Therefore, the ‘targeting-type’ neurons were activated either “when the direction difference between the retrieved location (co-location) and target direction was 0 degree” or “when the target directions were 90 and -90 degrees from the retrieved location.” The ‘targeting-type’ activity was observed more during the end of the background-cue period (Fig. 6c in the revised manuscript). These results suggest that the neurons with the “targeting-type” activity might represent the target location after the transference process. Using the same three-way nested ANOVA as for the definition of co-location, background and target-selective neurons, we defined the ‘targeting-type’ neurons as neurons showing a significant effect only for the target effect in the last time-bin (i.e., the 8th bin, 700-1000 ms) during the background-cue period.

Yes, “the 'transferring-type' neurons were activated only when the target directions were 90 and -90 degrees from the retrieved location.” The ‘transferring-type’ activity appeared earlier relative to the ‘targeting-type’ activity during the background-cue period (Fig. 6 in the revised manuscript). These results suggest the involvement of the ‘transferring-type’ activity in the transformation process from the retrieved location to the target location rather than the maintenance of it. Because the ‘transferring-type’ activity was transient during the background-cue period, we first determined a 300 ms of time-bin centering the peak time of the average response to the preferred target location in trials with 90 and -90-degree background-cues for each target-selective neuron. Then, we defined the ‘transferring-type’ neurons as the target-selective neurons showing significantly stronger responses to the preferred target location in trials with 90 and -90-degree background-cues than those with 0-degree background-cue (*P* < 0.05, t-test, one-tailed).

When we analyzed the relationship between the preferred co-locations during the item-cue period and the preferred target locations during the background-cue period, our data set included both the ‘transferring-type’ and ‘targeting-type’ neurons. The ‘transferring-type’ or ‘targeting-type’ can be determined independently of the activity-pattern (e.g., preferred co-location) during the item-cue period.

According to the reviewer’s suggestion, we added explanations of the ‘targeting-type’ and ‘transferring-type’ of neurons in more comprehensive ways with statistical analysis at single-neuron level and added numbers of these neurons in S1 Table in the revised manuscript.

***Comment 2:*** Fig 3a, b. The spike density functions (top) do not match the results of the F-values (middle) and the firing rates (bottom). For example, SDF shows almost no firing activity in the trials of the item II-AB, but the bar graph shows high firing rates in Fig 3a. It seems that the SDFs of Fig 3a and b were swapped by mistake.

***Response***: Thank you for pointing out the mistake. We corrected them in the revised manuscript (Figs. 4a&b).

**Responses to Reviewer #3:**

We thank the reviewer for the recognition of our work as well as the comprehensive comments on the description of our manuscript. We addressed all the comments listed point-by-point and revised our manuscript accordingly.

***Major comments***

***Comment 1:*** Up to my understanding, the long-term declarative memory mentioned in this paper is the spatially-associated memory between item-cue and background-cue. Because two different spatial coordinates were employed in the CMP task, i.e., allocentric coordinate for localizing the item-cue's position within the background-cue; egocentric coordinate for localizing the position of saccadic target, it will be easier for readers to catch up the logic of the task design if authors explicitly describe the exact meaning of item associated location.

***Response***: Yes, the reviewer is right. We revised the manuscript according to the reviewer’s suggestion (Page 6, Lines 90–91 & 100-101; Page 16, Lines 329-332).

***Comment 2:*** A key argument of this paper is that, after the item-cue (I-cue) onset, the activity of HPC neurons represents the retrieved item-location association memory. However, the results of signal neuronal activity (fig 2a) and population neuronal activity (fig 2c) show significant difference between the optimal and pair items, which does not support above argument. Since these two items share the same location, the neuronal response to these items should be similar if it really represents the memorized item associated location.

***Response***: Thank you for the critical and useful comment. We collected the item-selective neurons and examined the effect of item-location association memory on their item-selective responses. Figure 2 in the original manuscript (Fig. 2a-d, revised manuscript) showed the significant item-location association memory effect. However, some HPC neurons signaled only item identity, which caused a relatively large difference in population responses between the optimal items and their paired items (Fig. 2c). We therefore collected the item-selective neurons with large co-location index (*r* > 0.6, 109 of 136 item-selective neurons) to examine whether their responses to the optimal items and their paired items were similar or not by calculating values of area under curve (AUC) in the receiver operating characteristic (ROC) analysis. The results showed that the neurons with large co-location index could not discriminate the optimal items from the paired co-location items (*P* = 0.64) while they discriminated these best co-location items from other co-locations items (*P* < 0.0001) (Fig. 2g, revised manuscript). These results suggest that around 80% of the item-selective neurons in the HPC responded similarly to the optimal and paired items, suggesting that they really represented the memorized item associated location. We added figures 2e-g and the descriptions for ROC analysis in the revised manuscript (Page 8, Lines 143-158; Page 26, Lines 548-563).

In the revised manuscript, we also examined the item-location association memory during the background-cue period and found that the item-selective neurons showed a larger item-location association effect (*P* < 0.01) during the background-cue period compared with the item-cue period (Fig. 3c, revised manuscript). Moreover, ROC analysis suggested the representations of the memorized item associated location during the background-cue period even though all item-selective neurons were included in the analysis (Fig. 3d). We added figures 3 and the descriptions in the revised manuscript (Page 9, Lines 159-178).

***Comment 3:*** When looking up the results of fig 2d, there is a subset of hippocampal neurons showing the high correlation coefficient activity between optimal item and its pair item. Suggesting authors to analyze the response of these neurons to the optimal and pair items to see whether the activity is similar.

***Response***: We analyzed the subset as we responded to comment 2 above.

***Comment 4:*** The CMP task is difficult. To understand the electrophysiology data properly, I would like to suggest authors to present data of monkeys' behavior performance in the results section.

***Response***: We presented the behavioral data in the result section of revised the manuscript according to the reviewer’s suggestion (Page 6, Lines 108-115). In addition, we showed the training duration in each learning step of the CMP task for each animal (S3 Table)

***Minor comments***

***Comment 1:*** Line 27-29, the words of "south" "north" "turn left" mix the concept of allocentric and egocentric spatial frames. Need to describe these terms clearly.

***Response***: We revised the manuscript according to the reviewer’s suggestion (Page 3, Lines 41–42).

***Comment 2:*** Line 32, it is not clearly defined the meaning of "cognitive map". If I understand correctly, the "cognitive map" here means "allocentric map".

***Response***: The ‘cognitive map’ here means the spatial relationship of the environment in mind, which was represented in allocentric coordinate. We added an explanation of the cognitive map according to the reviewer’s comment in the revised manuscript (Page 3, Line 46).

***Comment 3:*** Line 41-42, I am not sure the description is correct. Please refer to the paper of Hasegawa et al., 2000 Science.

***Response***: We believe it is correct in the context of present study (construction process based on long-term declarative memory for remembering past and imagining future). However, we removed the original lines 41-42 from the introduction because we did not test it directly in the present study. We clarified the context of present study by contrasting to Hasegawa et al., 2000 Science (representation of past and future performance level during the single recording session) in the discussion of the revised manuscript (Page 20, Lines 421-425).

***Comment 4:*** In the caption of fig 2, the subtitle of panel a and c is not suitable because "item-selective neuron" is not consistent with "co-location" neuron in the text.

***Response***: The caption of fig. 2 was correct. However, the relationship between “item-selective neuron” and “co-location” neuron was not clear in the original manuscript. We clarified this point in the revised manuscript (Page 9, Lines 169-174).

***Comment 5:*** Line 112-113, authors wrote "This neuron showed the strongest response across all the co-location when the orientation of the background-cue was 90, while........". However, in fig 3a it seems that, in II-A/B, the data of the background-cue with 90-degree orientation is not significant higher than background-cue with 0-degree orientation.

***Response***: The description in the original manuscript was not accurate. We corrected it as “This neuron showed the strong responses across all the co-locations when the orientation of the background-cue was either 90° or 0°, while it showed only negligible responses when the orientation was -90°.” in the revised manuscript (Page 10, Lines 182-185).

***Comment 6:*** In fig 3, the raster plot of panel a and panel b might be miss-displaced, please have a check.

***Response***: We are sorry that the SDFs of the original Fig 3a and b were swapped by mistake. We corrected it in the revised manuscript (Figs. 4a and b).

***Comment 7:*** In fig 3, 4 and 5, the term "I-cue" and "B-cue" were not defined in both text and figure caption. In the results section of text, there is no description of what data are represented by the three curves (yellow, grey and black) in fig 3a-b, as well as in fig 4a and fig 5a-b.

***Response***: We defined the "I-cue" and "B-cue" in the original fig. 3 in the revised manuscript (caption of fig. 4, revised manuscript). This revised caption was referred to by the following figure captions (original figs. 4 and 5). We also described that the yellow, grey and black curves represented co-location, background and target effects estimated by F-values of the three-way nested ANOVA with those effects as main factors in the result section of the revised manuscript (Page 10, Lines 185-188 & 195-197; Page 13, Lines 257-259 & 263-264).

***Comment 8:*** Line 162, under this subtitle authors show neurons responded to specific target location in fig 5. Are there neurons response to 4 target locations, respectively?

***Response***: Yes, there were neurons responding to 4 target locations in each animal regardless of the recording hemisphere of each animal. We described this information with a new table summarizing a number of neurons with each target location as the best target location in the revised manuscript (S2 Table).

***Comment 9:*** Authors need to explicitly discuss the advantage of the present study (task design, etc.) in study of memory flexibility, comparing with previous studies.

***Response***: According to the reviewer’s suggestion, we described the advantage of the present study explicitly in the revised manuscript (Page 16, Lines 325-332; Page 17, Lines 342-346 & 353-357).

**Responses to Reviewer #4:**

We thank the reviewer for the recognition of our work as well as the important comments on the analysis or description of our manuscript. We addressed all the comments listed point-by-point and revised our manuscript accordingly.

***Major comments***

***Comment 1:*** The authors frame the paper around the use of memory in a flexible manner, and how this occurs at a single neuron level. The notion of "convergence" or neurons demonstrating selectivity based on a combination or conjunction of cues is commonly queried in hippocampal neurons, so this property was expected (although, it could be motivated based on prior literature in the Introduction, this literature is considered only in the Discussion). However, I found the notion and use of "transference" to be confusing/unclear and not well-motivated or explained in the Introduction. This made it difficult to interpret this property when presented in the Results. It seems like this response property of neurons emerged as an artifact of how the animals were trained and therefore may not be a general feature of how the hippocampus flexibly integrates information; it likely reflects a form of rotation performed during this task. It would be helpful to explain this up front in the manuscript, given that substantial space is devoted to it.

***Response***: Thank you for the good suggestion. According to the reviewer’s suggestion, we added motivations to investigate the ‘convergence’ and ‘transference’ properties in the introduction of the revised manuscript (Page 5, Line 72–81) (see Response to Comment 2 for details).

***Comment 2:*** Related to the above point, I wonder how general the authors think their findings are (the demonstration of 3 main types of neurons showing 'convergence', 'transference', and 'targeting'), or whether the specific types of neurons found here are strongly tied to the specific task and the amount of time needed to train animals to perform this kind of task. For example, is there an over-representation of 'transference' neurons simply due to the considerable training on the task and the manner in which the animals were trained (which is already evident in the 'transference' neurons), or is this a general feature of an episodic memory system? It is clear that the authors favor the later interpretation (third paragraph of the discussion), but a broader discussion of whether all of the response properties seen here are related to episodic memory versus considerable training on a specific behavior task should be addressed.

***Response***: Thank you for the important comment. In the present study, we actually investigated the flexible use of ‘semantic-like memory’ [i.e., long-term item-location association in allocentric coordinate (relative to the background image)] rather than episodic memory. In contrast to episodic memory for a single event, semantic memory can be formed through repetitive trainings. However, the training procedure is important to prevent the animals from learning the task in a gradual manner like probabilistic learning which would be supported by the striatum or neocortical areas rather than the hippocampus (Bayley and Squire, 2002; Knowlton et al., 1996). This is why we used the preliminary stimulus set, which would be easier for the animals to learn the task rule compared with the main stimulus set for the test (S8 Fig). If we used only the main stimulus set, it would have taken much longer time (or unsuccessful) for the animals to learn the task (S3 Table). In this situation, the combination of the item-cue and the background-cue may be directly associated with the animal’s behavioral response without involvement of the MTL even though the animals could solve the task. We also avoided this direct association by increasing the number of combinations between the two cues. Together, our present findings (the demonstration of 3 main types of neurons showing 'convergence', 'transference', and 'targeting') seem to be general at least for the flexible use of sematic memory. In addition to the task design and training procedure, the neuronal data also supported the significance of the present findings because we found that the target-selective activity in the HPC predicted the animals’ behavior, which suggests an involvement of the MTL in the task, as well as that the retrieved memory signal and incoming perception converged on single neurons that held both signal contents, which was against the conjunctive representation implicating a direct linkage between one particular sequence of the two cues and on a particular target. We added these explanations in the revised manuscript (Page 4, Lines 66-70; Page 17, Lines 353-365; Page 23, Lines 459-464 & 472-476). As the reviewer knows well, it is very difficult to investigate episodic memory in animal study (particularly physiological study). We therefore challenged its system by investigating the flexible use of semantic memory rather than episodic memory itself and hypothesized that the three neuronal operations might be employed by episodic memory system beyond the usage of semantic memory (Page 21, Lines 425-431).

In the present research paradigm, it seems to be reasonable to find the transferring-type activity because it would be required for the transformation process from the convergent-type activity into the targeting-type activity. However, we agree with the reviewer about the influence of our training on the activity pattern of the transference process. Our initial training of the animals on the 0° background image might determine the default position of the background image at 0°, which might influence the response pattern of the transferring-type activity. For example, if we first trained the animal using -90° orientation of the background-cue, the transference neuron might have shown their responses to 0° and 90° of the background-cue. Together, although the detailed response pattern could be influenced by the task and training procedure, the presence of the transferring-type activity may be general when semantic memory is used in a flexible way. We clarified the points in the revised manuscript (Page 18, Lines 375-385).

***Comment 3:*** Several correlation analyses are presented in the manuscript (Figure 2, co-location indices, Fig. 3d, similarity of background tuning, and in Fig. 4). Although I understood the theoretical significance of the 'co-location index' (Fig. 2), I generally found these correlation measures to be confusing, because 1) the description of these analyses, i.e. the data going into each of the correlations was not clearly stated and 2) the theoretical significance of each of the different correlations could be much more clearly spelled out in the Results (at least in my reading for those shown in Figs. 3 and 4). Both should be clarified - otherwise these analyses could be completely lost on the reader (I had to expend considerable effort to try to figure out what was being described/shown). For example, it was not clear to me how the correlation for the co-location index shown in 2b (example neuron) and 2d (population) was computed. In the description of the analysis in the Methods (lines 377-382), the sets of data points that are being correlated with each other are not actually stated. Is this the correlation between the mean firing rate in response to items within a co-location pair, such as I-A and I-B, across individual time bins (the 8 relevant time bins) or across individual trials and time bins? Without knowing what the correlation is computed over (i.e. the correlation in response across trials, or the correlation over time bins of mean response), the reader has no sense of the data going into the correlation, so it makes it difficult to interpret. I inferred that the correlation is being computed across time bins using the mean firing rate over trials, between the two co-location stimuli. This is potentially concerning if there are a low number of data points (are all 8 time bins included, or just those with significant F-stats?). Again, this can be addressed by clearly describing the analysis procedure and indicating what the correlation is being computed between (lines 377-382, 397-398, 409-411). Similarly, the data points going into the correlations shown in Figs. 3b and 3d were not clear to me. Is this the correlation between mean responses across the 3 background orientations for the 'best' and 'second-best' co-location stimuli, such that three data points are going into the correlation? Note that additional schematics may be helpful in illustrating these analyses.

***Response***: According to the reviewer’s suggestion, we clarified the data structure into each of the three correlation analyses (Page 26, Lines 538-547; Page 28, Lines 574-579; Page 28, 590-597) and the theoretical significance (Page 7, Lines 126-131; Page 11, Lines 213-216 & 219-221; Page 12, Lines 241-248) in the revised manuscript. We also added or revised schematics illustrating the three correlation analyses in the revised manuscript (S4 Fig for co-location index, Fig. 4b for similarity of orientation tuning, and Fig. 5b for matching index).

We examined the retrieval signal for both original Fig. 2b (example neuron) and 2d (population) by calculating Pearson correlation for individual item-selective neurons between the responses to the co-location items {i.e., [*f* (I-A), …, *f* (IV-A)] and [*f* (I-B), …, *f* (IV-B)]} (4 pairs, d.f. = 2) (S4 Fig). Here, ‘*f* (I-A)’ indicates the mean response across trials with the ‘item I-A’ as an item-cue. We calculated the correlation coefficients for the time bins with significant *F*-stats separately, and averaged them (*Z*-transformed values) among the significant time bins for each neuron. We clarified these points in the revised manuscript (Page 26, Lines 538-547; S4 Fig).

Yes, three pairs of data points went into the similarity of orientation tuning shown in original Figs. 3b and 3d.. We clarified this point in the revised manuscript (Page 28, 574-579; Fig. 4b).

***Minor comments***

***Comment 1:*** The training performed prior to the recording sessions is described in the SI text. It is easy for the reader to miss this description - please reference this text in the Methods where training is mentioned (lines 318-320).

***Response***: According to the reviewer’s suggestion, we referred to the S1 text in the Methods section of the revised manuscript (Page 22, Line 444; Page 23, Lines 463 & 468).

***Comment 2:*** Please include a few more details regarding the training. It looks like there are several phases of training: training on 4 stimuli with a 0 degree background cue, training on 4 stimuli with varied background cues (from -90 to 90), and then training on the 8 stimuli which were actually used in the recording sessions. Please describe the approximate duration of training for each of these phases prior to the recording task- how long did it take for the animals to be trained in each of these phases? In the final part of training, when the animals were trained on the 8 stimuli used in the paper, were they again trained first with the 0 degree background cue and then introduced to the varying direction of the cue? Were they trained on all 8 stimuli at once? Please include these details.

***Response***: We noted the numbers of training sessions (days) for each phase of the trainings in the new S3 Table. The main 8 stimuli were grouped into set A and set B, which the animals were trained on separately (Fig. 1a). In each set, we trained the animals with 0 degree background-cue first, and then trained with varied background cues (from -90 to 90). We described these details in S1 Text (Page 2, Lines 29–38) and S3 Table of the revised manuscript.

***Comment 3:*** Three-way ANOVAs were performed across sliding windows to examine the effects of 'co-location', 'background' and 'target'. Yet only 3 of the 5 backgrounds were used in this analysis (-45 and 45 degrees were excluded). What is the rationale for removing 2/5 of the data from the analysis? I found this to be particularly confusing when trying to understand the data shown in Figs. 3-5, since the top panels show firing rates overtime for all 5 background directions, yet when the data are broken down by background direction in the panels below, only 3 directions are shown.

***Response***: We excluded -45° and 45° in the detection of task-related neurons using three-way ANOVA because -45° and 45° background-cues bring about different target positions (i.e., top, right, bottom, and left) with those from the -90°, 0°, and 90° background-cues (top-right, bottom-right, bottom-left, and top-left) (S3 Text, both original and revised manuscript). It means that the use of all five background directions would bring about a strong bias for the target location and this is not appropriate for the ANOVA. We added this explanation in the method section of the revised manuscript (Page 25, Lines 523-526).

We presented firing rates over time for all 5 background directions to show the robustness of our data (top panel) while we presented the average firing rates only for 3 directions to make it easier for readers to understand the relationship among co-location, background and target location, which was examined by the three-way nested ANOVA.

We included the trials with -45° and 45° background-cues during the recording session in order to increase the number of configuration patterns and to prevent the animals from linking a combination of the item-cue and the background-cue to the target location directly, although we did not use these trials in the main analyses. We added this explanation in the method section of the revised manuscript (Page 23, lines 472-476).

***Comment 4:*** On lines 127-129 it is stated that "After background-cue presentation…neurons…exhibited either co-location-selective activities (14%) or background-selective activities (14%)." Yet, these numbers conflict with the data shown in Fig. 3C - with ~7% of neurons showing each of these properties after the background cue is presented.

***Response***: Fig. 3c of the original manuscript showed an instantaneous proportion at each time point, while “lines 127-129” of the original manuscript indicated the proportion during the whole background-cue period. In the latter case, we gathered the neurons with significant effects at different time points. Because the label of y-axis (“Percentage of selective neurons”) in the original Fig. 3c was misleading, we changed it as “Percentage of neurons with selective activity” and clarified what the values mean in the revised manuscript (S5b Fig).

***Comment 5:*** To show 'convergence' of retrieved information with the background cue ('perceptual') information, the authors analyze orientation tuning for co-location selective neurons (in Fig. 3D, 66 neurons). Were the co-location-selective neurons (those showing a 'retrieval' signal) defined only from the time period after the item cue was presented (before the background cue was shown)? Please clarify - I could not find this in the Methods. This definition would provide the clearest evidence that neurons which initially signal retrieval also show selectivity to the background perceptual information.

***Response***: The co-location-selective neurons (66 neurons in Fig. 3D, original manuscript) were defined using their activities during only the background-cue period. We clarified this point more in the revised manuscript (Page 9, Lines 169-174; Page 11, Lines 209-212; Page 25, Lines 517-523).

***Comment 6:*** Related to the above point, i.e. demonstrating 'convergent' retrieval signals and perceptual ('background') signals within individual neurons, I wonder if similar results are obtained by simply looking at the intersection of neurons which show both co-location selective information and background-selective information (intersection of neurons shown the individual effects presented in Fig. 3D). If the proportion of neurons demonstrating both kinds of selectivity are greater than would be expected by chance, this is another way to demonstrate the presence of convergent information at the single neuron level (one main point of the paper).

***Response***: Yes, we agree with the reviewer. We found 32 neurons exhibiting both co-location-selective and background-selective activities, which was significantly (*P* < 0.0005, χ^2^-test) greater than the expected number (n = 9.6). We added this result and used it as a definition of the “convergence-type” of neurons in the revised manuscript (Page 11, Line 207-209, S1 Table).

**We added following new citations in the revised manuscript.**

28.	Haskins AL, Yonelinas AP, Quamme JR, Ranganath C. Perirhinal cortex supports encoding and familiarity-based recognition of novel associations. Neuron. 2008;59(4):554-60.

29.	Fujimichi R, Naya Y, Koyano KW, Takeda M, Takeuchi D, Miyashita Y. Unitized representation of paired objects in area 35 of the macaque perirhinal cortex. The European journal of neuroscience. 2010;32(4):659-67.

41.	Knowlton BJ, Mangels JA, Squire LR. A neostriatal habit learning system in humans. Science. 1996;273(5280):1399-402.

42.	Bayley PJ, Squire LR. Medial temporal lobe amnesia: Gradual acquisition of factual information by nondeclarative memory. The Journal of neuroscience : the official journal of the Society for Neuroscience. 2002;22(13):5741-8.

48.	Shepard RN, Metzler J. Mental rotation of three-dimensional objects. Science. 1971;171(3972):701-3.

49.	Lamm C, Windischberger C, Moser E, Bauer H. The functional role of dorso-lateral premotor cortex during mental rotation: an event-related fMRI study separating cognitive processing steps using a novel task paradigm. NeuroImage. 2007;36(4):1374-86.

50.	Hawes Z, Sokolowski HM, Ononye CB, Ansari D. Neural underpinnings of numerical and spatial cognition: An fMRI meta-analysis of brain regions associated with symbolic number, arithmetic, and mental rotation. Neuroscience and biobehavioral reviews. 2019;103:316-36.

51.	Bellmund JL, Deuker L, Navarro Schröder T, Doeller CF. Grid-cell representations in mental simulation. eLife. 2016;5.

58.	Chen H, Naya Y. Automatic encoding of a view-centered background image in the macaque temporal lobe. bioRxiv. 2020; https://doi.org/10.1101/2020.03.01.971507.

60.	Hasegawa RP, Blitz AM, Geller NL, Goldberg ME. Neurons in monkey prefrontal cortex that track past or predict future performance. Science. 2000;290(5497):1786-9.

66.	Britten KH, Shadlen MN, Newsome WT, Movshon JA. The analysis of visual motion: a comparison of neuronal and psychophysical performance. The Journal of neuroscience : the official journal of the Society for Neuroscience. 1992;12(12):4745-65.
