# Supplementary material for: Hippocampal cells integrate past memory and present perception for the future
Source: PLoS Biol. 2020 Nov 18;18(11):e3000876. doi: 10.1371/journal.pbio.3000876 (PMC7673575; doi:10.1371/journal.pbio.3000876)
Supplement: Attachment — Submitted filename: Response Yang Naya.docx [file pbio.3000876.s017.docx]

**PBIOLOGY-D-20-00920R2**

**Responses to Reviewer #4:**

We thank the reviewer for re-reviewing our manuscript. We addressed all the suggestions listed point-by-point and revised our manuscript accordingly.

***Suggestion 1:*** Line 143-4: "One critical concern here might be whether the association effect reflected the locations retrieved from the item-cues." The implication or tone of this sentence (that it is problematic or concerning that the co-location effect may reflect retrieval processes) does not match the rest of the paragraph - which does show that the co-location signal is most consistent with reflecting retrieval of a common location. It may be helpful to revise this sentence.

***Response***: According to the reviewer’s suggestion, we revised the part as “The item-location association effect, revealed by the co-location index using the Pearson correlation coefficient, suggests two possible response patterns in the HPC during the item-cue period: the neuronal responses representing the locations retrieved from the item-cues and those representing individual items which were modulated by the co-locations …” in the revised manuscript (Page 8, Lines 143-147).

***Comment 2:*** The schematic in the new Fig S7 is a nice addition that helps to clarify the examined memory signals and processes in this task. I'd suggest moving it to the main text/combining it with a figure in the main text so that more readers may see it.

***Response***: According to the reviewer’s suggestion, we moved it to the main text in the revised manuscript (new Fig. 8). In addition, we revised the figure as it would explain the discussion parts in the main text explicitly (Page 18, Lines 344-353; Page 19, 378-385) as well as make the key findings of the present study clearer (Page 17, Lines 320-330).
